# Father presence and resilience of Chinese adolescents in middle school: Psychological security and learning failure as mediators

**DOI:** 10.3389/fpsyg.2022.1042333

**Published:** 2023-01-09

**Authors:** Xueyan Wei, Miao Zhuang, Linfang Xue

**Affiliations:** School of Humanities, Jiangnan University, Wuxi, Jiangsu, China

**Keywords:** father presence, adolescent's resilience, psychological security, learning failure, mediator

## Abstract

**Introduction:**

This study aimed to explore the association between father presence and adolescent resilience and the mediating role of psychological security and learning failure. Examining the mediating effects of learning failure and the chain mediating effect of psychological security and learning failure elucidated the link between father presence and adolescent resilience.

**Methods:**

The present study conducted a questionnaire survey among Chinese middle school students on father presence, resilience, psychological security, and learning failure. The survey collected 626 valid responses.

**Results:**

The findings showed that father presence, psychological security, learning failure, and resilience were significantly positively correlated; father presence had a direct effect on adolescent resilience, and psychological security and learning failure both mediated the relationship between father presence and adolescent resilience; psychological security and learning failure served as chain mediators between father presence and adolescent resilience.

**Discussion:**

This study aimed to provide theoretical and practical insights into the field of family education.

## Introduction

Traditional Chinese culture attaches great importance to the father's crucial role in children's growth. However, fathers have not done so well in modern times. The contemporary Chinese family has been characterized by father absence. According to a survey on family education in Shanghai, the phenomenon of father absence has increased. In terms of daily care, the proportion of children whose lives are mainly taken care of by their fathers decreased from 12.2 % in 2005 to 9.6% in 2015. The division of family responsibilities in children's lives began to change from “maternal care mode” to “mother is the main, grandparents are auxiliary” mode. Fathers' responsibility for their children's education was 23.7% in 2015 compared to 30.2% in 2005. According to a report on Chinese Family Development (2015), Chinese fathers' involvement in their children's education and accompanying needs needs to be greatly improved. Therefore, Chinese society expects the father to shoulder childcare and children's education responsibilities. Families also expect father absence to turn into father presence, as the father's active attendance will benefit the children's mental and physical health.

Father presence has been described as “his psychological presence in the offspring” (Neufeldt and Guralnik, 1994). According to the Western definition, a father connotes being before the other, being at hand, being in attendance, and showing his existence (Neufeldt and Guralnik, 1994). Hence, the father's psychological presence signifies his psychological closeness and availability to the child (Krampe, 2009). Studies indicated that high-quality father presence contributes to various positive developmental results (Pruett et al., 2017), such as promoting healthy psychological development (Li et al., 2007). Meanwhile, father absence leads to poor adaptability and resilience (Li and Tian, 2018).

Scholars investigated whether a higher level of positive father presence leads to stronger resilience. Zhao (2021) asserted that, in China, father involvement could positively affect adolescents' mental health. Specifically, father involvement can increase problem-oriented coping, decrease emotion-oriented coping, and maintain children's mental health levels. Another study also alluded to the association of father involvement with psychological security (Yang and Zhang, 2016; Yin, 2016). As individuals with a strong learning orientation gain experience from failure (Yu et al., 2004), Chinese adolescents may be resilient when facing hardships. The aforementioned factors might lead to the development of resilience. However, no evidence has been collected either on the role of father presence in promoting resilience or on the interaction of factors during this process. Thus, we examined each of these issues in a sample of adolescents. As the saying goes in China, “born to raise, raise and teach, teach them properly.” We look forward to enlightening Chinese fathers on how to become “fathering-capable fathers.”

In the present study, our primary aim was to assess the effects of father presence on adolescents aged 12–18 years in the Chinese context. Based on the framework of resilience in action, we hypothesized that high-quality father presence, as an external resource, could directly affect adolescents' resilience. Further, it could directly induce a feeling of internal security. A strong father presence might help troubled adolescents solve problems through internalized learning failure. Thus, we hypothesized that learning failure and psychological security might mediate the association between father presence and adolescent resilience. Finally, father presence would affect adolescent resilience through the chain of mediators of psychological security and learning failure. We expected that our findings could help design interventions that promote the development of adolescents.

## Literature review

### Father presence and adolescent resilience

Based on research on father upbringing, we investigated the relationship between father presence and adolescent resilience. We defined father presence as follows: “fathers [being] involved in their children's cognition, emotion, and behaviors” (Gao et al., 2020). This concept contains seven dimensions: material insurance, emotional communication, guidance participation, future planning, teaching experience, model demonstration, and overall intentions (Krampe and Newton, 2006; Xue, 2019). We adopted the following definition of resilience: “the function of mental processes and behaviors in boosting personal assets and protecting individuals from the potentially negative effects of stressors” (Fletcher and Sarkar, 2013). The concept encompasses five dimensions: goal concentration, emotional control, positive cognition, family support, and interpersonal assistance (Hu and Gan, 2008).

A team of psychologists from scientific research institutions put forward a framework of resilience in action (using the Resilience and Youth Development Module. http://www.wested.org/, 2003). According to the model, resilience is an innate adolescent potential. Adolescents have psychological needs for safety, love, belonging, challenge, talent, and value during the development process. The satisfaction of these needs depends on protective factors or external resources from school, family, society, and peer groups. If external resources provide the psychological needs of adolescents, then adolescents will naturally develop individual characteristics that will constitute internal resources, including cooperation, empathy, problem-solving, and self-efficacy. These internal resources will protect adolescents from risk factors and promote healthy development.

Based on the aforementioned model, the presence of a father might be inferred to be related to adolescent resilience. While peers and circumstances may be equally influential, parents and family are unchanged elements in most young people's lives. Among adolescents, vulnerability and resilience largely depend on the family environment (Rutter, 1993). Studies confirmed the multiple psychological effects of father presence on children's growth and development, constituting a new perspective in father-child relationship research. Krampe and Newton (2006) pointed out that the level of father presence is of great concern for young individuals' psychological health and personality development. Good family function (e.g., conjugal/couple relationship, parent-child relationship, father involvement) is an important protective factor of individuals' resilience (Wright and Masten, 2005). In China, a large body of evidence also suggested that fathers are of great importance in developing children's intelligence, personality characteristics, sociality, and gender roles (Pu and Lu, 2008). Family elements, particularly those related to the father, are closely related to children's mental health.

Social learning theory (Bandura, 1991) helps elucidate “father presence.” It states that individuals' social behaviors are not generated by nature or instinct but are rather gradually formed through continuous observation, learning, imitation, and reinforcement. The essence of observational learning is imitation. For children, imitation is a crucial behavior acquisition, and parents are their primary objects of imitation. Parents' daily behavior, habits, and emotional attitudes have an essential impact on the growth and development of children. In the development of individual psychology or behaviors, a father's importance must not be ignored. Traditional Chinese culture attaches great importance to the father's care and upbringing of his children, namely, his presence. In the current Chinese society, owing to the rapid change and development of the social economy, fathers can achieve direct (e.g., daily care) and indirect interaction (e.g., *via* various media). This interactive parent–child mode could demonstrate multiple aspects associated with father presence. Hence, it might have a deep influence on adolescent resilience (***Hypothesis 1***).

### Psychological security

Psychological safety includes implicative, cognitive, and affective structures. As such, safety may be treated as a psychological phenomenon with a standard structure. Security can be viewed as a state of inner peace, confidence, a positive attitude, trust, subjective wellbeing, openness, and relaxation (Zotova and Karapetyan, 2018). Maslow et al. (1945) described psychological security as “a feeling of confidence, security, and freedom from fear and anxiety, particularly when one's present (and future) needs are met.” In the early Chinese studies of psychological security, security was defined as the emotion one experiences when escaping from a dangerous situation or being protected (Huang, 2004). Psychological security is a premonition of potential physical or psychological danger or risk, as well as a sense of power and powerlessness when handling affairs, mainly referring to the sense of certainty and control (Cong and An, 2004). We adopted the definition of psychological security proposed by Cong and An (2004): a good and stable psychological experience of teenagers in their study, family, daily lives, and social interactions.

Krampe and Newton (2006) argue that participation helps children coordinate with their fathers by helping them approach, recognize, and understand him and eventually embrace and internalize his influence. In this process, if the perceived quality of the parent–child relationship is good, then the children's security level is correspondingly high. The father plays an equally important role as the mother in forming the children's security (Zhang, 2012a). In contrast, according to Horney's (1950) theory of anxiety, growing up without the love of parents and the warmth of family creates insecurities. Meanwhile, resilience is remarkably positively correlated with the psychological security of left-behind children (namely, students with poor academic performance) (Xu et al., 2013). Hence, children's security is closely linked to their fathers and their resilience.

Yang and Tan (2007) found that the level of individual security is significantly correlated with family type, parental income, and the parent–child relationship. Yin (2016) considered that the dimension of physical interaction between junior high school students and their fathers could significantly predict their psychological security level. Wang (2009) reported that the father's parenting style is greatly correlated with the daughters' sense of security, among which emotional warmth and understanding are positively correlated with a sense of security.

Meanwhile, some studies found differences in social frustration in groups with high and low levels of psychological security. People with high levels demonstrate markedly reduced social frustration and increased resilience (Akhmadeeva and Galyautdinova, 2021). The effects of psychological security on an individual's social adaptation (Xu et al., 2013), self-esteem (Zeng et al., 2017), interpersonal relationship, interpersonal trust (Chen and Yin, 2018), and resilience (Wang and Zhang, 2018) have also been confirmed. Thus, we aimed to examine whether psychological security may exist in the association between father presence and adolescent resilience (***Hypothesis 2***).

### Learning failure

Politis (2005) argued that failure should be viewed as a personally cherished learning resource. Learning failure, as an internal force, is defined as “an individual learning from a failed experience.” It relies on the individuals to reflect on their thoughts and actions as a strategy to reduce the probability of future failure (Holger et al., 2011). Failure can create tremendous value and experience, and the learning behaviors after failure determine the acquisition of knowledge and experience (Wang et al., 2021).

The home is the best school for adolescents, and parents are the primary teachers. Experienced parents can help children develop different abilities, including learning from failure. Morgan (1894) introduced the concept of “trial and error” as a learning method. This topic has been explored in different areas to find effective ways to promote learners' improvement. According to the experiential learning circle theory, learning is a process of “starting from experience, then returning to experience, reforming or changing experience, and then creating knowledge” (Kolb, 1984). As adolescents grow, they are exposed to all kinds of life experiences, including their own, those taught by others, and successes and failures. These valuable resources can be transformed into an important driving force to promote an individual's growth and development based on rational utilization.

Edmomdson (1999) pointed out that a high level of psychological security, which is conducive to speaking out and expressing opinions freely, positively impacts learning failure. Tang et al. (2014) also found that psychological security could significantly predict an individual's positive learning failure. Furthermore, studies showed that people with higher learning orientations enjoy the experience of failure. Compared with those with less learning motivation, they may be more resilient when working hard (Yu et al., 2004). The active involvement of fathers in this learning process encourages the youth to take action to deal with failure—that is, a father's presence could indirectly promote resilience (Opondo et al., 2016). Therefore, a high-quality father's presence would positively impact adolescent resilience by fostering the latter's internal strengths of psychological security and learning failure. In this study, we aimed to examine whether father presence influences adolescent resilience *via* the mediator of learning failure (***Hypothesis 3***) and whether psychological security and learning failure play chain mediating roles in this association (***Hypothesis 4***).

## Research methodology

### Participants and procedure

The participants are Chinese students who studied in the four middle schools of Jiangsu Province and Shanxi Province. The cluster sampling method was used to choose some classes from the above schools for research. Information about students' perceptions of father presence, psychological security, learning failure, and resilience was voluntarily provided. The questionnaire included assurances of confidentiality and anonymity.

We distributed 720 questionnaires. After eliminating unusable ones (such as regular answers and answers with a missing rate of over 50%), 626 survey questionnaires were retained, with an answer rate of 86.9%. Junior high school students account for 58.5%, and senior high school students represent 41.5%. The students' average age was 14.89 years (SD = 2.17), ranging from 12 to 18. In our sample, there were 44.1% men, 55.9% women, 65.2% urban students, and 34.8% rural students.

### Materials

We employed four scales to measure the four variables mentioned above: father presence, adolescent resilience, psychological security, and learning failure. Answers were rated on a 5-point Likert scale, ranging from 1 (strongly disagree) to 5 (strongly agree).

#### Father presence scale in China

The father's presence was assessed using the scale compiled by Xue (2019). The scale contained seven dimensions: material insurance (α = 0.824), emotional communication (α = 0.909), participation in the guidance process (α = 0.829), future plans (α = 0.877), teaching experience (α = 0.916), model demonstration (α = 0.851), and overall intentions (α = 0.934). It included 38 items (e.g., “My father can meet my basic material needs in life and study”). The reliability of this scale, Cronbach's α, was 0.859.

#### Adolescent resilience scale

Adolescent resilience was measured by the scale developed by Hu and Gan (2008). The scale contained 27 items involving five dimensions: goal concentration (α = 0.904), emotional control (α = 0.876), positive cognition (α = 0.831), family support (α = 0.779), and interpersonal assistance (α = 0.889). Sample items included, “I like the course to be able to stimulate curiosity, even if it may be difficult to learn.” Cronbach's α for this scale was 0.879.

#### Psychological security scale

Psychological security was measured using the scale developed by Cong and An (2004). The scale consisted of the following two dimensions: interpersonal security (α = 0.846) and certainty in control (α = 0.857). The scale contained 16 items (e.g., “I feel that life is full of uncertainty and unpredictability”). The reliability of this scale, Cronbach's α, was 0.914.

#### Learning failure scale

Adolescents' failure to learn was measured by the scale of Xue (2019). The scale contained 25 items and four dimensions: failure cognition (α = 0.866), reflective analysis (α = 0.841), experience transformation (α = 0.880), and prudent attempt (α = 0.919). Sample items included, “I had to figure out what was causing my failure.” Cronbach's α was 0.920.

### Data analysis

We ran SPSS version 21.0 for descriptive statistics, correlation analysis, and regression analysis, and AMOS version 17.0 for mediating analysis.

### Common method variance

Based on the index data of Williams and McGonagle (2016), we tested for common method variance bias with a one-factor test. The results stated a total variance of 10%, with a threshold of < 25%, indicating that our study did not have a problem with common method variance.

The specific analysis proceeded as follows. We ascertained the maximum explanation rate of the factors, with consideration for the reference value of 40%. A rate lower than 40% indicated the absence of significant common method bias in the study. We conducted an unrotated exploratory factor analysis on a total of 21 research variables. The results indicated that four factors had characteristic roots >1, which explained 65.58% of the total variance. The variance explanation rate of the first factor was 24.65%. Therefore, the data in this study were less affected by the common method bias.

## Data analysis and results

### Descriptive and correlational analysis

Descriptive statistics and correlations among the studied variables are reported in Table 1.

**Table 1:** Descriptive statistics and correlations among study variables (*N* = 626).

**Variables**	**M**	**SD**	**1**	**2**	**3**	**4**	**5**	**6**
1. Gender	1.52	0.28	1					
2. Age	14.89	2.16	0.35	1				
3. Father's presence	145.39	33.23	0.07	−0.12*	1			
4. Psychological security	53.42	14.09	0.13	−0.09*	0.50***	1		
5. Failure to learn	98.03	16.19	0.12	0.26*	0.33***	0.33**	1	
6. Resilience	96.27	16.36	0.28*	0.15	0.43**	0.63***	0.55***	1

*p < 0.05; **p < 0.01; ***p < 0.001.

Father presence, psychological security, learning failure, and adolescent resilience were significantly correlated. Specifically, the father's presence positively affected psychological security, learning failure, and adolescent resilience. Higher levels of father presence, psychological security, and learning failure ability were related to a higher level of adolescent resilience.

***Aim 1: Does Father Presence Directly Affect Adolescent Resilience?***

We used Mplus software (version 7.0) to conduct a mediating effect. The independent variable was father presence, while the adolescent's resilience was the dependent variable.

**Figure 1:**
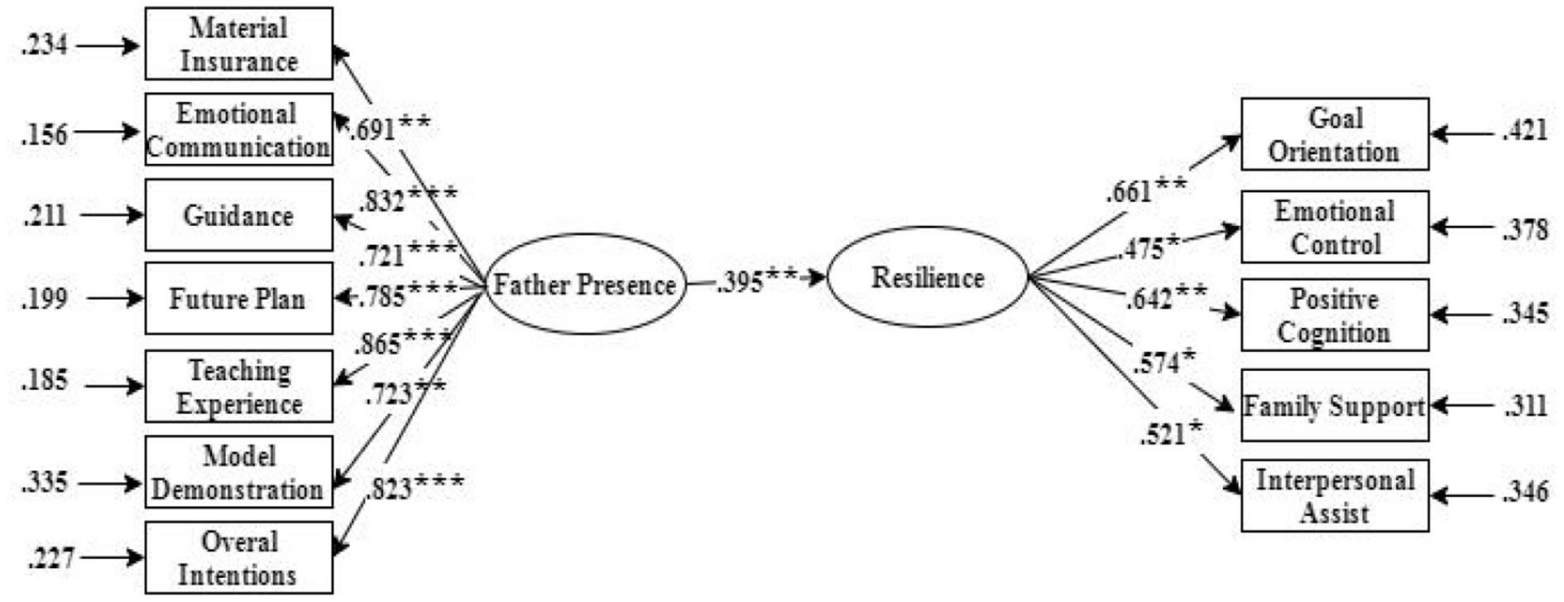
Model of direct effect of father presence on resilience. *p < 0.05; **p < 0.01; ***p < 0.001.

χ^2^*/df* = 3.582, RMSEA = 0.067, CFI = 0.924, TLI = 0.909, SRMR = 0.065, The normalized path coefficient C presented in this study was significant, indicating that the x → y path was significant; that is, father presence significantly positively predicted the resilience (β = 0.397, *P* < 0.001). The data indicated that the direct effect of father presence and resilience was significant (Figure 1).

***Aim 2: Does Father Presence Affect Adolescents' Resilience via the Mediator's*** ***Psychological Security?***

We used Mplus software (version 7.0) to conduct a mediating effect. The independent variable was father presence, and the dependent variable was the adolescent's resilience. At the same time, psychological security was an intermediary variable.

**Figure 2:**
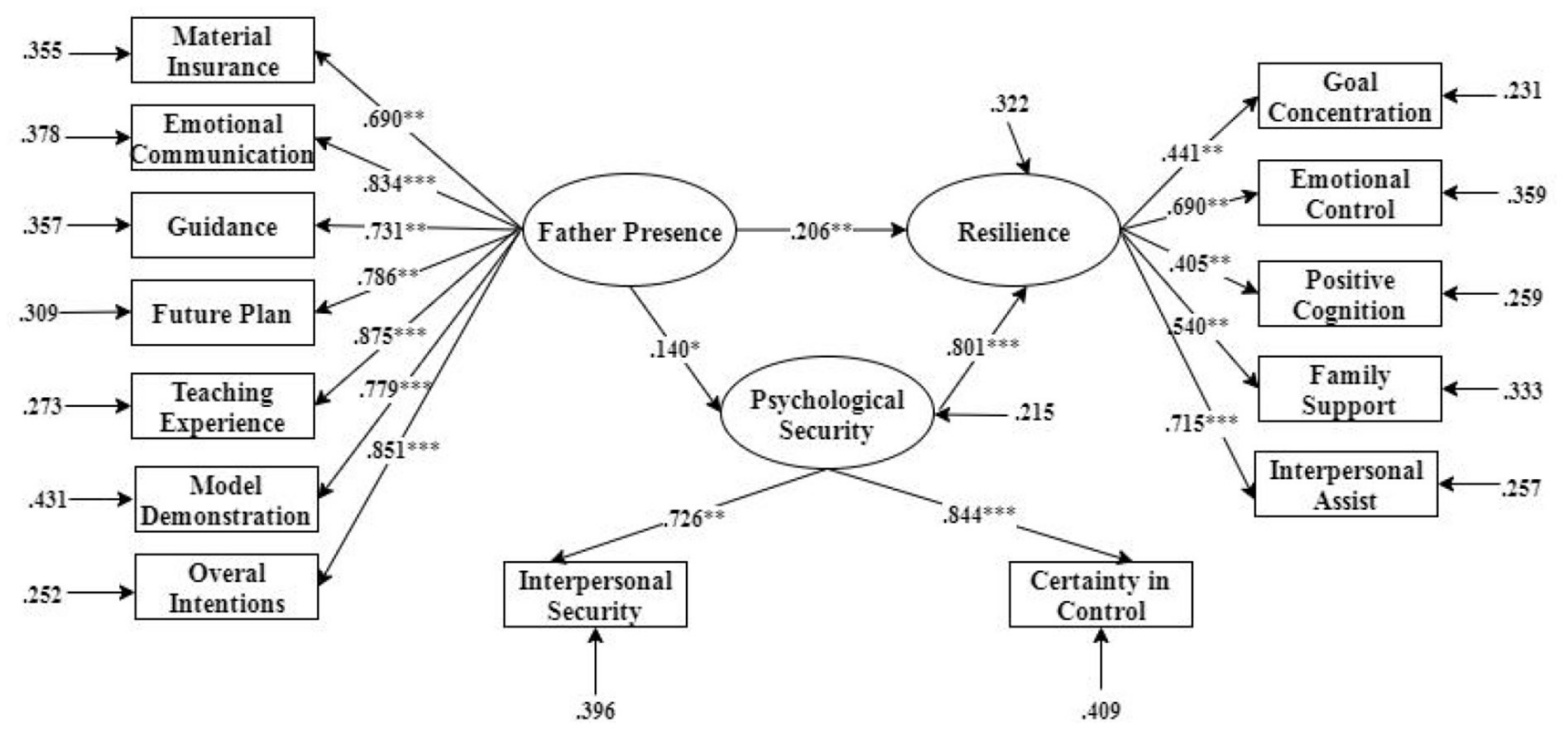
Mediating model of psychological security between father presence and resilience. *p < 0.05; **p < 0.01; ***p < 0.001.

χ^2^*/df* = 3.728, *RMSEA* = 0.077, *CFI* = 0.923, *TLI* = 0.929, *SRMR* = 0.062, the data stated that psychological security served as a mediator between father presence and resilience, with a mediating effect of 0.112 (Figure 2).

***Aim 3: Does Father Presence Affect Adolescents' Resilience via the*** ***Mediator-Learning failure?***

Mplus 7.0 (version 7.0) software was used in our study to conduct a mediating effect. The independent variable was father presence, and the dependent variable was the adolescent's resilience. Conversely, learning failure was an intermediary variable.

**Figure 3:**
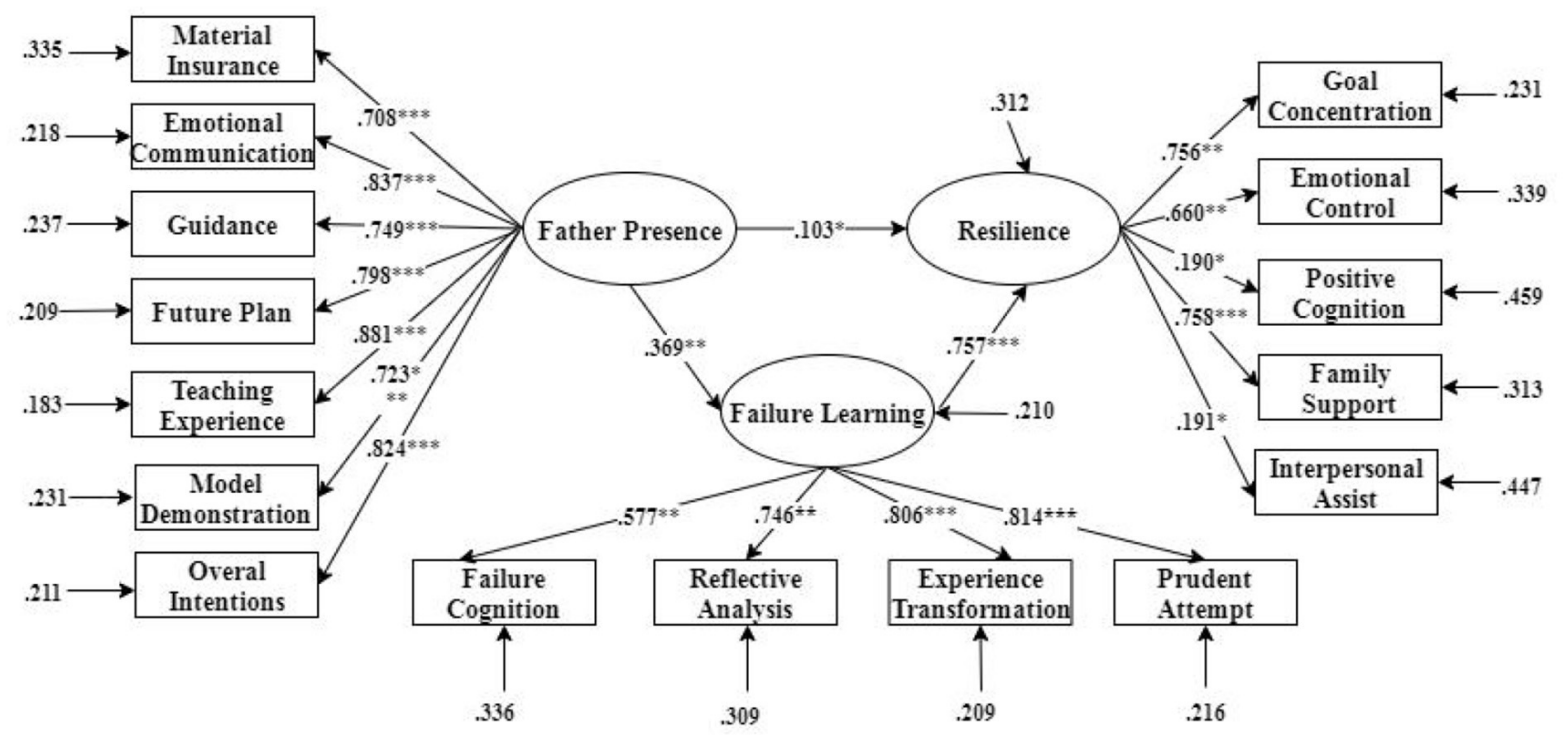
Mediating model of learning failure between father presence and resilience. *p < 0.05; **p < 0.01; ***p < 0.001.

χ^2^/df = 2.736, RMSEA = 0.055, CFI = 0.932, TLI = 0.944, SRMR = 0.031, the data indicated that learning failure severed as a mediator between father presence and resilience, with a mediating effect of 0.279 (Figure 3).

***Aim 4: Do Psychological Security and Learning failure Play the Chain*** ***Mediating Roles in the Association Between Father Presence and Adolescent*** ***Resilience?***

The bootstrap method was used to further test the significance of the mediation model. The results showed that psychological security significantly mediated the relationship between father presence and resilience (95% CI = 0.004–0.063) and that interpersonal security significantly mediated the relationship between father presence and resilience (99% CI = 0.022–0.071).

As examined in the previous section, learning failure and psychological security are simple mediators between father presence and adolescents' resilience. A correlation analysis showed a significant correlation between learning failure and psychological security (*r* = 0.312^***^, *p < * 0.01). Psychological security and learning failure were included as mediators in the structural equation model to discover the chain effect of psychological security and learning failure on father presence and adolescent resilience.

**Figure 4:**
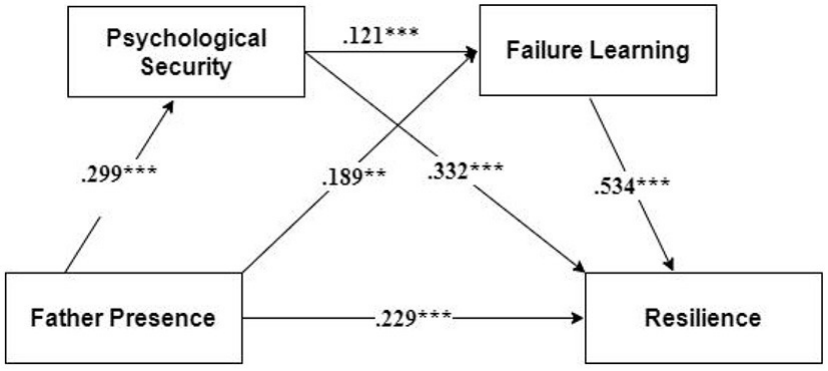
Chained mediation model with psychological security and learning failure as mediators (Model 1). **p < 0.01; ***p < 0.001.

χ^2^*/df* = 2.938, RMSEA = 0.055, CFI = 0.955, TLI = 0.927, SRMR = 0.055. The above indicators were up to standard, and the model fit well. It showed that psychological security and learning failure mediate between father presence and resilience, and a chain mediating role model was obtained (Figure 4).

According to the path coefficients of Model 1, father presence significantly positively predicted resilience (β = 0.229, *P* < 0.001), psychological security (β = 0.299, *P* < 0.001), and learning failure (β = 0.189, *P* < 0.01). Psychological security observably predicted learning failure (β = 0.121, *P* < 0.05) and resilience (β = 0.227, *P* < 0.001). Learning failure remarkably positively predicted resilience (β = 0.227, *P* < 0.001).

The 95% confidence interval was further calculated, and the results are shown in Table 2. The confidence intervals for the direct effect of father presence on resilience and the mediating effect of father presence on resilience through psychological security and learning failure, respectively, did not include 0; hence, the mediating effect was established.

**Table 2:** Chain mediating effect analysis of psychological security and learning failure (N = 626).

**Path effect**		**95% Confidence Interval**		**Indirect effect value**	**Effect of the amount**
		***Boot CI* Upper limit**	***Boot CI* Lower limit**		
Direct effect	Father's presence- resilience	0.048	0.112	0.231	
Mediating effect	Father presence-psychological security- resilience	0.005	0.055	0.12	26.1%
	Father presence-learning failure- resilience	0.033	0.068	0.045	11.5%
	Father presence-psychological security-learning failure- resilience	0.002	0.008	0.007	2.3%
	Overall mediating effect			0.172	39.9%
Overall effect				0.403	

## Discussion and conclusions

### Findings

We explored the relationship between father presence, psychological security, learning failure, and adolescent resilience in the context of contemporary society in China. Our analysis showed the direct and indirect paths among the concepts.

#### Direct path: Father presence and adolescent resilience

When testing whether external assets such as father presence can promote resilience development, we found that resilience tended to be stronger as the level of father presence increased. This is consistent with the results of the study by Pu et al. (2012a) and Wu et al. (2017). Pu et al. (2012b) reported a significant correlation between father presence and college students' resilience. Wu et al. (2017) also confirmed that the level of father presence has a significant predictive effect on left-behind teenagers' resilience. Zakeri et al. (2010) found that parents' active involvement is significantly positively correlated with children's resilience. Zhang (2012b) examined the sub-dimensions of parenting styles in a sample of high school students and found correlations with resilience: the father's emotional warmth and overprotection have positive predictive effects on high school students' resilience, whereas the father's rejection has negative predictive effects.

According to the resilience framework in action, individuals can build their resilience through external factors. Father's presence provides adolescents with material insurance and emotional communication, among others, to satisfy their needs. Hence, adolescents' internal resilience can be greatly developed.

#### The indirect path from father presence to adolescent resilience

Next, we examined whether external assets, such as father presence, could promote resilience development *via* psychological security and learning failure. We proved the mechanism of influence of internal resources on resilience (see Figure 4 and Table 2).

##### Psychological security

We found a strong mediating path between father presence, psychological security, and resilience (see Table 2). According to the theory of anxiety, adolescents attach great importance to their parents' concern and love, which offer them strong psychological security and support to cope with difficulties. Xu et al. (2013) noted that a good family relationship is crucial for individuals to establish a sense of security. A positive and harmonious parent–child relationship represents parents' acceptance of and intimacy with their children, affecting the latter's daily communication, boosting their confidence in dealing with interpersonal relationships, and improving their sense of security. Meanwhile, a good sense of interpersonal security translates into positive external support that helps children deal with risk factors. Thus, the high-quality parent–child relationship represented by the father's presence can give individuals a higher sense of security in interpersonal communication. Their sense of safety allows them to draw on support from others and to adjust effectively in the face of challenges, which allows for greater adaptation.

##### Learning failure

Learning failure played an important mediating role. According to experiential learning circle theory (Kolb, 1984), the rich experience and knowledge from the father and positive interpersonal support can help the individual be more proactive in the next attempt after failure and obtain better problem-solving abilities and adaptability. The impact of learning failure in domestic settings has received scant attention compared to its prominence in the business world. We focused on how father presence influenced adolescent resilience through learning failure. As such, our work expanded the application of learning failure to family research based on experiential learning circle theory.

As an important companion and guide in individual growth, the father is also an important transmitter of individual life experience. High-quality father presence represents the transmission of rich life experience, through whom adolescents can gain plenty of knowledge, experience, and spiritual resources, apart from material security. This learning process leads to better failure-coping abilities and indirectly cultivates resilience (Niobe and Gillman, 2000; Thomas et al., 2008).

##### Psychological security and learning failure

Regarding the path of psychological security and learning failure, we identified the mechanism through which father presence affected resilience through the aforementioned key mediating factors. Both of the mediators were internal resources. Under the driving force of the father's presence, psychological security, a kind of inner feeling of support, transferred confidence and courage for learning failure, an outward-oriented internal resource. Both psychological security and learning failure supported the development of resilience.

Hershenberg et al. (2011) found that American adolescents (89% Caucasian) in close family relationships have high levels of security and positive behavior. Moreover, Van Ryzin and Leve (2012) reported that children (88% Euro-American, 7% mixed ethnic background, 2% Hispanic, 1% African American, 1% Native American, and 1% Asian American) with a good sense of security are more likely to be liked by their peers, get positive responses from their interactions with peers, and have better emotional adjustment abilities. Therefore, positive family resources may lead to beneficial results.

As a unique positive family resource, a high-quality father's presence can help build a harmonious and open family atmosphere. On the one hand, individuals can feel warmth and security while growing up and form a high level of secure attachment. On the other hand, father presence can address individuals' problems and mistakes with an open and inclusive mind, fostering positive externalization behavior. Meanwhile, psychological security has a significant positive effect on learning failure (Tang et al., 2014). With the improvement of individuals' security, for one thing, they can fully express their attitudes, feelings, and opinions without too much concern when talking about their own failures and defects. For another, in a high-quality parent–child relationship, individuals can also obtain more guidance from their fathers, promoting individuals' reflection and the formation of a positive attitude toward failure. In turn, the ability to analyze and solve problems is enhanced, contributing to the improvement of resilience. The chain effect of psychological security and learning failure verified the influence of father presence on adolescent resilience through chain mediation and the internal mechanism of the influence of father presence on adolescent resilience.

### Limitations

Our study had some limitations, which may provide directions for future research. First, in the context of Chinese culture, this study focused on the impact of Chinese fathers on adolescent resilience. We did not compare Chinese fathers with those in Western countries. Future research can explore different fathers from different cultural backgrounds, such as those who express concern and love and their influence on adolescent resilience.

Second, we did not explore the role of mother presence on adolescent resilience. Mothers offer support for adolescents, but whether the role of the mother is different from that of the father merits in-depth investigation. Thus, future research can examine the functional differences between fathers and mothers in the family and whether fathers and mothers produce different influences on children's resilience development.

Finally, our research tools had certain limitations. In addition to the youth resilience and psychological security scales, we used other scales that we developed, compiled, and revised based on their suitability with respect to our research objectives. The adaptability of the questionnaire was confirmed by cross-sectional tests; however, additional time and follow-up research are needed to ensure suitability for broader samples.

### Conclusions and implications

The data indicated that father presence, as an external influencing factor, affected resilience in the sample of Chinese adolescents. The high-quality presence of fathers greatly benefited the sample adolescents' psychological development. Our study's results confirmed the father's role in the family as an important external support for Chinese adolescents (Pu et al., 2011).

Our findings also affirmed the mediator role of psychological security and learning failure in the association between Chinese father presence and adolescent resilience. Our study underlined the function of psychological security and learning failure in promoting the resilience of adolescents facing trouble. When an individual encounters stress or challenges, they can appropriately interact with the environment. The internal resilience factors (e.g., cognitive, emotional, mental, physical, and behavioral) come into play to help the individual cope with maladjustment. According to attachment theory, when an individual experiences secure attachment, they can interact effectively and as desired with the environment (Cassidy, 1999). Moreover, failure could serve as a learning tool, which creates experiences and drives individuals to attempt something repeatedly (Andrew et al., 2021). Thus, fathers and their children could benefit from the failure of their adolescents.

We expected to contribute realistic and profoundly significant insights to the field of family education.

## Data availability statement

The raw data supporting the conclusions of this article will be made available by the authors, without undue reservation.

## Ethics statement

The studies involving human participants were reviewed and approved by Jiangnan University. Written informed consent to participate in this study was provided by the participants' legal guardian/next of kin.

## Author contributions

All authors listed have made a substantial, direct, and intellectual contribution to the work and approved it for publication.
